# The experiences of mental healthcare users regarding the quality of their major depressive disorder treatment

**DOI:** 10.4102/hsag.v30i0.2896

**Published:** 2025-07-07

**Authors:** Frans M. Lekoloane, Marang T. Mamahlodi

**Affiliations:** 1 Department of Health Studies, College of Human Sciences, University of South Africa, Pretoria, South Africa

**Keywords:** major depressive disorder, mental healthcare users, treatment, experiences and perceptions

## Abstract

**Background:**

Millions of people worldwide are affected by major depressive disorder (MDD). However, those with MDD do not receive treatment. More studies have been conducted on the efficacy of medications rather than the subjective experiences of mental healthcare users (MHCUs).

**Aim:**

This study was conducted to investigate the experiences of MHCUs regarding the quality of their MDD treatment and to suggest recommendations that are necessary to bring a positive change towards the quality of MDD treatment.

**Setting:**

The study was conducted at a Tshwane District Hospital.

**Methods:**

A qualitative, exploratory study was used in this study. Seventeen MHCUs were selected using purposive sampling. Data were collected using observations, interviews and focus group discussions, and the data were analysed using grounded theory.

**Results:**

One theme emerged from this study: ‘The holistic treatment approach’. Two sub-themes emerged: ‘Administration of service delivery’ and ‘Challenges of service delivery’.

**Conclusion:**

Research findings show that the current treatment of MDD is beneficial. However, challenges exist that negatively affect the quality of the MDD treatment and they need to be addressed urgently by relevant stakeholders.

**Contribution:**

The study could contribute through: (1) providing recommendations that, if implemented, could assist the decision-makers and MHCPs in understanding the quality of MDD treatment from the MHCUs’ perspective and utilise these for policy development and to make necessary treatment adjustments.

## Introduction

Major depressive disorder (MDD), known as depression, is a common psychiatric disorder (Idris et al. 2023:1). It presents with symptoms such as low mood, diminished interest in pleasurable activities, weight changes, appetite changes, disturbed sleeping patterns, fatigue, feelings of worthlessness, poor concentration and suicidal ideation (Idris et al. 2023:1).

For the diagnosis of MDD to be made, at least four of the symptoms mentioned should be experienced, with each episode lasting for at least 2 weeks (Tu et al. 2023:1069). Major depressive disorder needs to be treated because, when it is untreated, it can reduce the quality of life and contribute towards increased mortality rates (Rousseau et al. 2021).

Major depressive disorder is an overwhelming burden to public health (Proudman, Greenberg & Nellesen 2021:619). Globally, millions of people suffer from MDD (Idris et al. 2023:1).

The estimates of the World Health Organization (WHO) suggest that by 2030, MDD could be a leading burden of disease (Nady et al. 2023:14). Recent literature supports these estimates as they show an increase in the prevalence of MDD (Alwis, Baminiwatta & Chandradasa 2024:368).

The prevalence of MDD in South Africa is also on the rise, as one in six South Africans are likely to present with MDD symptoms (Booysen, Mahe-Poyo & Grant 2021:1). The rise in MDD prevalence can be attributed to factors such as increased unemployment rates, increased crime rates, coronavirus disease 2019 (COVID-19) and other stressful events (Qubekile, Paruk & Paruk 2022:2).

However, many South Africans with MDD do not receive treatment because of factors such as inadequate resources, policy discrepancies and underinvestment by the government in mental health treatment (Booysen et al. 2021:1). The mentioned factors affect the quality of the MDD treatment.

Treatment refers to the provision of comprehensive care, management and rehabilitation of mental health care users with MDD (National Department of Health 2023:13). The reasonable quality treatment of MDD consists of professionals such as specialist psychiatrists, psychiatric nurses, occupational therapists, clinical psychologists and social workers (Uche 2023:1). The common MDD treatment includes but is not limited to pharmacotherapy, cognitive behavioural therapy, psychotherapy and supportive therapy (Tu et al. 2023:1069). The MDD treatment can be provided through hospitalisation and outpatient programmes as per the mental healthcare users’ (MHCUs’) needs (Sachs et al. 2023:1).

Research studies show that the treatment quality of MDD is inadequate (Hassem & Laher 2022:2; Matseke 2023:71). Most studies focus on the efficacy of pharmacological treatments (Copa et al. 2024:61). There are scarce studies on the subjective experiences of MHCUs regarding the quality of their MDD treatment, which shows the novelty of this research study. This research study provides data regarding the quality of MDD treatment from MHCUs’ perspective.

Although researchers and mental healthcare providers (MHCPs) are expert professionals, MHCUs are stakeholders in the MDD treatment, and therefore their subjective views need to be established to contribute to positive change in the quality of their MDD treatment (Renger, Macaskill & Naylor 2020:4). The importance of exploring the experiences of MHCUs is supported by various authors, such as Idris et al. (2023:1) and Mvunelo, Haffejee and Thandar (2024:1).

This study is significant as it establishes the experiences of MHCUs regarding the quality of their MDD treatment, which could contribute to the research and body of knowledge in public health studies. The study could contribute through: (1) providing recommendations that, if implemented, could assist the decision-makers and MHCPs in understanding the quality of MDD treatment from the MHCUs’ perspective and utilise these for policy development and to make necessary treatment adjustments.

### Aim of the study

The aim of the study is to investigate the experiences of MHCUs regarding the quality of their MDD treatment and to suggest recommendations that are necessary to bring a positive change towards the quality of MDD treatment.

### Research objectives

The following objectives are formulated to meet the aim of the study:

- To explore the experiences of MHCUs regarding the quality of their MDD treatment.
- To suggest recommendations that are necessary to bring a positive change in the quality of MDD treatment.

## Research methods and design

A qualitative research approach and an exploratory research design were undertaken to assist the researcher in exploring and establishing the MHCUs’ experiences and to achieve ‘a deeper understanding’ of the experiences of MHCUs regarding the quality of their MDD treatment (Ugwu & Eze 2023:20). A constructivist grounded theory was applied to collect and analyse the qualitative data and to understand the experiences of MHCUs with MDD regarding the quality of their MDD treatment (Mohajan & Mohajan 2023:2).

### Study setting

A Tshwane District Hospital was selected because it has a high admission of MHCUs. This hospital is considered one of the largest hospitals in Gauteng, South Africa, in terms of infrastructure and beds available for MHCUs. This hospital provides services such as psychiatric management, psychotherapy, occupational therapy, social work and others. It has male and female general adult psychiatric wards and outpatient services, among others. It is also affiliated with universities such as Sefako Makgatho Health Sciences University.

### Study population

Adult MHCUs diagnosed with MDD as a primary diagnosis were selected. The ages varied between 22 years and 56 years during the time the study was conducted. The MHCUs received their MDD treatment in Tshwane District Hospital as either inpatients or outpatients.

### Eligible criteria

Eligible criteria include the following: The MHCUs receiving MDD treatment at Tshwane District Hospital, and the MHCUs who did not show psychotic features and were able to give consent to participate in the study.

### Exclusion criteria

All MHCUs with other psychiatric disorders were excluded in the study. Those MHCUs who did not give consent were also excluded.

### Sampling and sample size

A non-probability sampling technique known as purposive sampling was used to select the participants who satisfied the research’s objectives as deemed so by the researcher (Obilor 2023:4). Seventeen participants were interviewed, and data saturation was reached (Sosa-Díaz & Valverde-Berrocoso 2022:2).

### Recruitment of mental healthcare users

The services of an operational manager were utilised to assist with identifying the MHCUs who met the inclusion criteria because the operational manager had access to MHCUs’ files. The operational manager signed a third-party confidentiality agreement form.

The outpatients who were potential participants were recruited while waiting to see their MHCPs. This was done to avoid destabilising the daily routine at the department. Potential participants were given a participant information sheet with research-related information to read while waiting for their turn to be seen by the MHCPs. The researcher explained the purpose of the research as plain as possible and provided clarity where necessary. The potential participants were asked to be interviewed during their next appointments. The potential participants who came back for their appointments and agreed to participate in the study were requested to sign a consent form and an agreement to be recorded form before the interviews.

Potential participants who were inpatients were also recruited, and they were given a participant information sheet to read, and clarity was provided where necessary. The inpatients were recruited when they were resting with no sessions to attend. The potential participants who agreed to participate in the study were requested to sign informed consent forms and agreement to be recorded forms. The inpatient participants were interviewed the following day.

### Data collection methods, procedure and management

Data collection commenced after obtaining an Ethical Clearance Certificate from the university and after obtaining permission from the study setting management. The researcher also liaised with the Head of Department and Operations Manager and thereafter data collection commenced.

Observations, interviews and focus group discussions were data collection methods used in this research study. Observation was performed first, interviews followed, and thereafter, the focus group discussions. An interview schedule form was used for interviews. A focus group discussions schedule form was used for focus group discussions.

The researcher conducted a pilot study to test the research instruments and to make necessary adjustments (Poto-Rapudi 2021:72). A pilot study was necessary to evaluate the practicalities of the main research study (Poto-Rapudi 2021:72). Three participants took part in the pilot study and adjustments were made to the interview schedule.

The unstructured observations were made at the study setting and during interviews to understand the natural setting of the participants and to interpret non-verbal cues observed to establish the thinking patterns of the participants. The participants gave written consent to observation.

Informed consent forms, confidentiality forms, agreement to be recorded forms, ethical forms and other forms that may contain biographical information of participants and sensitive information were placed in a locked cupboard at the researcher’s home, and they will be shredded after 5 years. All interviews and focus groups discussions were recorded with the permission of the participants using a mobile phone voice recorder, and the recordings were deleted after being transferred into the USB using a laptop, which was password protected. The field notes, transcriptions, data analysis and interpretations were safely placed in a locked cupboard. Recordings will be deleted and transcriptions shredded after 5 years.

### Data analysis

Data analysis in the grounded theory approach uses constant comparative analysis, which is a procedure for coding and category development and it involves evaluating and comparing data across categories and then patterns are identified (Mohajan & Mohajan 2022:2–3, 8). Data analysis commenced after interviewing four participants. Data collection and data analysis occurred simultaneously (Mohajan & Mohajan 2022:8). There are three main stages of data analysis in grounded theory: open coding, axial coding and selective coding (Phillips, Tichavakunda & Sedaghat 2023:3).

Three data sets, namely observations, interviews and focus group discussions, were integrated during data analysis. Observations field notes were compared with transcriptions of interviews and of focus group discussions. The themes and/or categories emerged from the three sets of data were compared. Similar themes and/or categories were grouped together and questions emerged as guided by the research problem, and the questions were added to the interview schedule and were used in the next interviews.

#### Open coding

The first four interviews’ recordings were transcribed manually. Line-by-line coding was used, and similar data were grouped from all four transcriptions (Mohajan & Mohajan 2022:10). Similar data were categorised and compared to field notes from observations. The categorisations led to more questions, which were added to the existing interview schedule so that the researcher does not lose track of the research problem.

The four more interviews were conducted to address emerged questions, and one focus group was conducted with the same four participants. The transcriptions were analysed and similar data were grouped. The categorised data were compared to field notes from observations. More categorisations led to more questions, which were added to the interview schedule.

Another four interviews were conducted and one more focus group discussion was conducted. Transcriptions were made from the interviews and focus group discussions. The transcriptions were analysed and similar data were grouped and categorisations were made, which led to more questions.

Five more interviews were conducted as one more participant who met the eligibility criteria volunteered to participate. A third focus group was not conducted because the participants did not consent to it. The five interviews were transcribed and analysed, and similar data were categorised and compared to existing categories.

The data from the extra five interviews did not produce new categories, and therefore, data saturation was reached and data collection discontinued (Sosa-Díaz & Valverde-Berrocoso 2022:2).

#### Axial coding

The categorisations that emerged during open coding were compared. Similar categorisations were regrouped and themes were developed. The developed themes were compared to the data to determine whether the themes were the true reflection of the data gathered.

#### Selective coding

The last step of data analysis in grounded theory is selective coding (Phillips et al. 2023:3). The themes that emerged during axial coding were compared and new themes were developed. The emerged themes are the theoretical codes of the study. The theoretical codes were compared with transcribed data, categorisations and themes to establish the connections, and an abstract theory was developed to explain the experiences of the MHCUs regarding the quality of their MDD treatment.

### Ensuring rigour (trustworthiness)

The researcher ensured the credibility of the research findings. The researcher managed to build rapport through informal conversations with participants in their own languages. Interviews with inpatients were conducted 2 days later, and with outpatients, during their next appointments. The researcher used an interview schedule as a guideline, and more questions were derived from participants’ responses, and participants were made aware of that before the interviews. Three data sets, various data sources (inpatients and outpatients) and various investigators were used to attain triangulation (Zelĉãne & Pipere 2023:4). The researcher noted their own interpretations and shared them with participants after interviews for comment to ensure that the interpretations represented the correct opinions of the participants (Humphreys et al. 2021:857).

The transcriptions, the field notes, the data analysis steps and any information related to the research were safely stored so that a repetition of this study with similar participants in a similar context will yield similar findings (Ogutogullari 2022:13). A detailed description of the research problem and research findings has been provided, and therefore, the research findings could be transferred to different settings (Megheirkouni & Moir 2023:849). The services of a qualitative research assistant and external auditor were utilised to assist with confirmability. The researcher provided them with all the information pertaining to the study and they signed the third-party confidentiality agreement forms.

### Ethical considerations

Ethical clearance to conduct this study was obtained from the University of South Africa, Human Science Ethics Committee (Reference no: 46963774_CREC_CHS_2023). The researcher liaised with the Head of the Department of Psychiatry and Operations Management prior to the commencement of data collection.

The researcher provided the participants with a written participant information sheet that explains the purpose of the study, the processes to be followed and the role of the participants. The researcher further explained the purpose of the study orally, using the languages of participants for maximum comprehension.

The participants were assured that they were not coerced to participate in the study and that they had the right to refuse and to pull out of the study at any point in time, and in the event they felt coerced, they should report to the operations manager. An informed consent form was developed and signed by participants before commencement of interviews.

Participants’ personal information was not divulged to anyone as guided by the *Protection of Personal Information Act* (POPI Act) (Raaf, Rothwell & Wynne 2022:280), and they were immediately referred to as ‘Participant A, B, C, etc.’ They were assured that the information provided was for research purposes only.

The researcher tested the interview questions during the pilot study and feedback was requested from the participants to ensure that the interview questions were not upsetting, degrading and/or stigmatising. The participants were encouraged to consult with the psychiatric nurse for debriefing and to report any discomfort caused by the researcher’s questions (Olum 2022:5).

## Results

The demographic information of participants is presented in Table 1. The theme, sub-themes and categories are presented in Table 2.

**TABLE 1:** Participants’ demographic information.

Age (years)	Number of participants	Gender	Race
20–30	8	Male = 4 Female = 4	African people = 6 Mixed race people = 2
31–40	4	Male = 2 Female = 2	African people = 4
41–50	1	Male = 0 Female = 1	African people = 1
51–60	4	Male = 0 Female = 4	African people = 4

**TABLE 2:** List of theme, sub-themes and categories.

Themes	Sub-themes	Categories
1. The holistic treatment approach	1.1. Administration of service delivery	1.1.1. Multidisciplinary team 1.1.2. Providing helpful treatment
	1.2. Challenges of service delivery	1.2.1. Shortage of MHCPs 1.2.2. Lack of urgency 1.2.3. Infrequency of therapy sessions 1.2.4. Negative attitudes of MHCPs

MHCPs, mental healthcare providers.

## Participants’ demographic information

Seventeen MHCUs participated in this study. Male participants were six, whereas female participants were 11. The age groups of the participants were between the ages of 22 years and 56 years. Out of 17 participants, 10 were outpatients and seven were inpatients.

### Theme 1: The holistic treatment approach

The participants reported that the quality of MDD treatment should have a holistic approach. A holistic approach involves collaboration of health professionals to address presenting problems. However, in this study, the holistic approach was reported to be inadequate. This is what participants had to say:

‘I have been to the social workers with children and a partner. They have been very helpful.’ (Participant 6, 56, Female, 4 years)

‘Family therapy [*is needed*] … because some of them [*family members*] do not understand our sicknesses.’ (Participant 2, 24, Female, 2 years)

‘My husband does not want to understand … he is suggesting that I stop drinking medications because when I am on medication, I do not have interest for intimacy … they have been calling him for sessions but he is not interested.’ (Participant 7, 53, Female, 5 years)

‘[*W*]e do not have a support system outside. When we are discharged, we are only depending on the hospital and then when I read some other countries, they have got support system for people who are depressed where they can go and ventilate their feelings, they share.’ (Participant 13, 53, Female, 24 years)

#### Sub-theme 1.1. Administration of service delivery

The quality of MDD treatment should involve reasonable administrative-related services. Administrative-related services such as hospitalisation, follow-ups, allocation of health professionals and others should be adequate. However, the study’s findings show that there were administrative-related challenges, which negatively affect the quality of MDD treatment:

‘I stayed in casualty for 20 days and there were no beds in the ward. There were many of us without beds … If there was one bed available then they would take one, if there were two beds available then they would take two just like that.’ (Participant 10, 34, Female, 2 months)

‘It takes a while to get follow-up appointments with them [*mental health care practitioners*].’ (Participant 5, 29, Male, 7 months)

‘[*S*]ince admission is my second week, I am still waiting for the social worker. I do not know whether he or she will still come and the psychologist … the sister said, the social worker is still busy because there are a lot of patients in the hospital, so I am still in the queue waiting for him or her to see me.’ (Participant 14, 51, Female, 1 year 8 months)

**Category 1.1.1. Multidisciplinary team:** Participants reported to have attended various therapy interventions conducted by various health professionals for MDD treatment. The therapy interventions included but were not limited to pharmacotherapy, psychotherapy, occupational therapy, nursing care, physiotherapy and social work. This is what the participants had to say:

‘After the conversation I had with the GP then he concluded that I might have depression. That is why he referred me to a psychologist.’ (Participant 1, 37, Female, 2 years)

‘[*T*]he nurses are helping us with everything like issuing the treatment [*medication*], the OT [*occupational therapist*] helping us with everything that we need, the social workers as well, they are with us [*helping us*], the doctors [*are helping us*] …’ (Participant 4, 34, Male, 11 years)

‘I attended occupational therapy, to assess my functioning level because at work they also wanted a report on how I am functioning …’ (Participant 13, 53, Female, 24 years)

‘I will say being depressed is not a death sentence, through the help of the social workers, psychologists and even going to the OTs [*occupational therapists*] and in the ward, being actively involved if there are games in the ward or through the interviews, it helps a lot, it alleviates things that are on my mind.’ (Participant 14, 51, Female, 1 year 8 months)

**Category 1.1.2. Providing helpful treatment:** Most participants (*n* = 16) reported that the current MDD treatment was helpful. This is what they had to say:

‘[*T*]he treatment that I have been getting in this hospital has been okay so far. No complaints … I am happy with the treatment.’ (Participant 1, 37, Female, 2 years)

‘The treatment of depression in this hospital is okay. I am happy with the treatment that I am receiving. It helps me a lot.’ (Participant 7, 53, Female, 5 years)

‘The treatment of depression is okay. I cannot complain. The treatment is working for me. I had headaches, muscle tension, panic attacks and I feel better now. It makes me feel better.’ (Participant 9, 27, Female, 6 months)

‘I will say being depressed is not a death sentence, through the help of the social workers, psychologists and even going to the OTs [*occupational therapists*] and in the ward, being actively involved if there are games in the ward or through the interviews, it helps a lot, it alleviates things that are on my mind.’ (Participant 14, 51, Female, 1 year 8 months)

#### Sub-theme 1.2. Challenges of service delivery

The participants in this study revealed concerns regarding the administration of mental health services. Access to mental health services is inadequate, which was visible when a participant from Ekurhuleni district visited Tshwane district for mental health services:

‘[*A*]nd I am staying far I do not even know if I can make it to the appointment [*Participant 8 is from Tembisa*]. It has been difficult for me to get help where I am from. My problem is the distance.’ (Participant 8, 22, Female, 2 months)

The infrastructure inadequacies were also concerning to the participants. Infrastructure inadequacies contributed to overcrowding and inadequate mental healthcare services provision, which affects the quality of MDD treatment negatively:

‘They must expand the facility, renew the infrastructure, more [*physical*] space for therapy …’ (Participant 3, 27, Male, 1 year 8 months)

‘I stayed in casualty for 20 days and there were no beds in the ward. There were many of us without beds … If there was one bed available then they would take one, if there were two beds available then they would take two just like that.’ (Participant 10, 34, Female, 2 months)

‘It is so challenging. Because I am not staying in the ward where we are having patients with depression, we are just mixed. Some they are praying; some they are singing and I do not want noise.’ (Participant 13, 53, Female, 24 years)

**Category 1.2.1. Shortage of mental healthcare providers:** The participants showed concerns with the shortage and availability of MHCPs. This is what they had to say:

‘It would be nice if there were more psychologists and more OTs [*occupational therapists*] at the hospitals. I think they are short-staffed. More psychologists and more OTs will be helpful in public institutions.’ (Participant 1, 37, Female, 2 years)

‘In my observation, the hospital is short-staffed. I do not know if the doctors do not want to come to the wards or they are too busy but I believe they are short-staffed.’ (Participant 5, 29, Male, 7 months)

‘I will suggest that there should be more psychiatric nurses hired and who are skilled …’ (Participant 14, 51, Female, 1 year 8 months)

**Category 1.2.2. A lack of urgency:** Participants are concerned with the lack of urgency in the treatment of MDD. They felt that the rate at which services are provided is slow.

Participant 3 puts it this way:

‘[*T*]hings tend to be slow.’ (Participant 3, 27, Male, 1 year 8 months)

Other participants said:

‘It takes longer for us to get appointments.’ (Participant 1, 37, Female, 2 years)

‘We wait a long time before being seen by the doctor.’ (Participant 2, 24, Female, 2 years)

‘It takes a while to get appointment follow-up appointments with them [*mental health care practitioners*].’ (Participant 5, 29, Male, 7 months)

‘[*S*]ince admission is my second week, I am still waiting for the social worker. I do not know whether he or she will still come and the psychologist …’ (Participant 14, 51, Female, 1 year 8 months)

**Category 1.2.3. Infrequency of therapy sessions:** The participants are concerned with the frequency with which they have been attended during their hospitalisation. They felt that they had not been attended to as regularly as possible. This is what they had to say:

‘… We only have sessions, I think, once or twice a week. More sessions will be helpful.’ (Participant 1, 37, Female, 2 years)

‘More sessions are needed. Sometimes we can stay the whole week without attending a therapy session, which is not good.’ (Participant 5, 29, Male, 7 months)

‘We rarely attend sessions, since I came here, I attended one [*1*] individual session by the OT [*occupational therapy*] and one [*1*] group by the OT [*occupational therapy*] and I think that is why we stay long in the hospital.’ (Participant 6, 56, Female, 4 years)

**Category 1.2.4. Negative attitudes of mental healthcare providers:** Participants showed concerns with the behaviours of some MHCPs towards them. They felt that those MHCPs were giving them a negative attitude. This is what they had to say:

‘I am not happy with the way the nurses treat us. They disrespect us. The way they talk to us is not nice. We are undermined. And they lose our documents and blame us for losing them.’ (Participant 7, 53, Female, 5 years)

‘[*T*]he way the staff addresses us is not good, it is like they are disrespecting us, and we came here because we are sick and we need help and it does not mean we are useless people, it is not right.’ (Participant 6, 56, Female, 4 years)

‘I have realised that 90% of the staff in the ward do not know anything about the mental health care users they are just here for the paycheque so if there would be enough staff that are trained it is going to be easier for them to treat us and for us to comply to the treatment …’ (Participant 14, 51, Female, 1 year 8 months)

‘The treatment is fine is just that they must exercise patient’s right. You must see other patients’ point of view before jumping to conclusion …’ (Participant 16, 28, Male, 4 weeks)

## Discussion

The aim of this research study was to investigate the experiences of MHCUs regarding the quality of their MDD treatment.

### Sub-theme 1.1. Administration of service delivery

A good quality of MDD treatment should be beneficial to the MHCUs. The MDD treatment is considered beneficial when it can alleviate the presenting signs and symptoms of MDD and restore maximum functioning (Alva 2023:521). The study’s findings show that the current treatment of MDD has been beneficial to the MHCUs to some extent.

#### Category 1.1.1. Multidisciplinary team

This study also shows that a good quality of MDD treatment should have a consistent holistic approach (National Department of Health 2023:9). A holistic approach to MDD treatment refers to consideration of all spheres of lives of participants, their interactions and how they relate to each other (Stebletsova & Scanlan 2023:40).

A holistic approach involves the collaboration of multidisciplinary team interventions consisting of specialities such as pharmacotherapy, psychotherapy, occupational therapy and social work, among others (Uche 2023:1).

Major depressive disorder has the potential to negatively affect various areas of MHCUs’ lives, which requires the active involvement of various health professionals. When there is active involvement of a multidisciplinary team, challenges such as stigma can be alleviated through services such as family integration (Kapadia 2023:857).

The treatment of MDD with pharmacotherapy as the first line of treatment is well documented; however, this study shows that the quality of MDD treatment requires the involvement of various treatments, which require various health professionals. This collaboration of various health professionals in providing quality MDD treatment is supported by the study of Tadmon & Olfson (2022:11).

The study’s findings show that the holistic approach was inadequate in the current MDD treatment. Some participants believed some of their challenges, which contributed to their MDD diagnosis were not properly addressed. This shows that an inadequate holistic approach may negatively affect the quality of life of the MHCUs.

Some of the challenges affecting a holistic approach to MDD treatment are administrative-related challenges, which were reported in this study. For the good quality of MDD treatment to be provided, there should be adequate administration of mental health services. The mental health services administration, such as accessibility, consultation, hospitalisation, follow-ups, allocation of health professionals and others, should be reasonable.

However, the issues of accessibility of mental health services were evident in this study. The South African Government advocates for the usage of primary healthcare settings such as clinics for the improvement of accessibility and provision of mental health services (Kathree et al. 2023:1262). However, this study shows that the primary healthcare settings are currently not adequately equipped for the provision of mental health services (Stein et al. 2022:395). This was visible when some MHCUs had to travel from one district to the other for mental healthcare services.

### Sub-theme 1.2. Challenges of service delivery

The recruitment and/or the allocation of MHCPs needs to be adequate, as it was visible in this study. The shortage of MHCPs is well documented and cannot be ignored, as it forms the foundation of the inadequate quality of MDD treatment (Hassem & Laher 2022:2). Factors such as inadequate budgets by the government, a lack of interest in mental health and universities not producing enough MHCPs contribute to the shortage of MHCPs in the treatment of MDD (Booysen et al. 2021:2).

The shortage of MHCPs was acknowledged by the Minister of Health, Dr MJ Phaahla, who suggested alleviation of the shortage of MHCPs through: (1) providing mental healthcare training and skills development and (2) budgeting for contracted MHCPs (Minister of Health 2023:7).

Participants in this study also raised concerns with the lack of urgency in the treatment of MDD. The lack of urgency in the treatment of MDD has been supported by the studies of Alva (2023:521) and Marx et al. (2023:2). The contributing factors to a lack of urgency could be attributed to the shortage of MHCPs, the complexity of MDD, the response by MHCUs to therapy and guidelines of MDD treatment (Alva 2023:521; Marx et al. 2023:2). The lack of urgency shows the need for advanced treatment modalities for quicker remission (Alva 2023:522).

There were concerns regarding the frequency of therapy sessions from participants. They felt that MHCPs do not attend to them as frequently as reasonably possible. This led to uncertainties for participants, as they wondered whether their issues would be attended to or not. Infrequent therapy sessions could be attributed to issues such as the shortage of the MHCPs, among others (Matseke 2023:71). Nevertheless, the quality of MDD treatment requires consistent therapy sessions (Tseng et al. 2023:14).

There were also concerns regarding the negative attitudes of the MHCPs towards the MHCUs. Negative attitudes of MHCPs towards MHCUs are well documented and concerning (McKenzie, Gregory & Hogg 2022:2; Peñuela-O’Brien et al. 2023:176). The relevant decision-makers need to address this challenge because it could contribute to defaulting on treatment and not seeking treatment, which could be detrimental to the mental health of participants.

## Limitations of the study

The study focused on the experiences of MHCUs with MDD as a diagnosis only. The research results do not represent the experiences of MHCUs with other psychiatric disorders. The participants may have been cautious with their responses as they were at the hospital. At home, a different setting, they could have provided more information.

## Conclusion

This study investigated the experiences of MHCUs regarding the quality of their MDD treatment. The study’s findings were significant in addressing the existing research gap on investigating the subjective experiences of MHCUs. It has managed to provide data regarding the quality of MDD treatment from MHCUs’ perspective.

The research findings show that the current treatment of MDD is beneficial, as it alleviates the presenting signs and symptoms of MDD and restores maximum functioning. The MDD treatment is more beneficial with the active involvement of a multidisciplinary team.

However, challenges exist, such as the shortage of MHCPs, a lack of urgency, infrequency of therapy sessions and negative attitude of MHCPs, which negatively affect the quality of the MDD treatment.

The following recommendations stem from the research findings.

### Mental healthcare providers

Mental healthcare providers with an interest in mental health are needed. Mental healthcare providers should avail themselves as frequently as possible to render mental health services. The MHCPs should advocate for holistic treatment through consistent involvement of a multidisciplinary team to promote total recovery and to promote provision of good quality treatment.

### Decision-makers

The relevant decision-makers should address administrative challenges raised in this study, such as shortage of MHCPs, a lack of urgency, infrequency of therapy sessions and negative attitude of MHCPs. The relevant decision-makers should increase the current ratio of MHCPs towards MHCUs through increased recruitment of MHCPs. They should advance the processes of improving access to mental health services, such as improving the standards of primary settings to cater to and manage MHCUs with mental illnesses such as MDD. The decision-makers should consider putting strict measures in place to address the negative attitude of MHCPs towards MHCUs.
